# Development and Validation of Machine Learning Models for Predicting Early Cognitive Decline Using Home Sensor–Derived Behavioral Data: Sensors in-Home for Elder Wellbeing (SINEW) Cohort Study

**DOI:** 10.2196/79490

**Published:** 2026-04-01

**Authors:** James Patrick Moon, Khalid Abdul Jabbar, Tony Chin Ian Tay, Laura Tay, Rathi Mahendran, Tze Pin Ng, Shian Ming Tan, Wilbur Zhi Hao Koh, Weng Yan Ying, Ah-Hwee Tan, Tih-Shih Lee, Iris Rawtaer

**Affiliations:** 1 Department of Psychiatry, Sengkang General Hospital, Singapore, Singapore; 2 Research Office, Sengkang General Hospital, 110 Sengkang E Way, Singapore, 544886, Singapore, 65 69302288; 3 Department of Geriatric Medicine, Sengkang General Hospital, Singapore, Singapore; 4 Mind Care Clinic @ SBF, Mind Science Centre, National University of Singapore, Singapore, Singapore; 5 Population Health and Integrated Care Office, Sengkang General Hospital, Singapore, Singapore; 6 Department of Psychology, Sengkang General Hospital, Singapore, Singapore; 7 School of Computing and Information Systems, Singapore Management University, Singapore, Singapore; 8 Laboratory of Neurobehavioral Genomics, Neuroscience and Behavioral Disorders Programme, Duke-NUS Graduate Medical School, Singapore, Singapore; 9 SingHealth Duke-NUS Academic Medical Centre, Singapore, Singapore

**Keywords:** dementia, neurocognitive disorder, mild cognitive disorder, prefrailty, frailty, internet of things, aged, technology

## Abstract

**Background:**

As the global population continues to age, the prevalence of geriatric conditions, including dementia and frailty, is also increasing. Early identification of individuals at an elevated risk of these conditions, such as those presenting with mild cognitive impairment (MCI) or prefrailty, can provide a critical window for prompt intervention aimed at preventing or reversing disease progression. To promote such early identification, there is a burgeoning interest in the use of digital sensor technology and predictive modeling.

**Objective:**

This study aimed to use a continuous, home-based monitoring sensor system for older adults to distinguish those exhibiting normal aging from those with MCI, early dementia, prefrailty, or frailty, and to predict their transition from normal aging to one of these conditions.

**Methods:**

This longitudinal cohort study will recruit 200 community-dwelling adults aged ≥65 years with normal cognition or MCI at baseline. A multi-sensor system will be installed in participants’ homes, including passive infrared motion sensors, door contact sensors, bed sensors, medication box sensors, wearable activity bands, and Bluetooth proximity beacons. These devices will continuously capture spatiotemporal activity patterns, mobility indicators, sleep behaviors, and medication-taking routines. Annual assessments will include standardized cognitive tests (eg, Montreal Cognitive Assessment, Mini-Mental State Examination, Rey Auditory-Verbal Learning Test, digit span, Color Trails Test, semantic fluency, Stroop), frailty measures (modified Fried phenotype, gait speed, grip strength), mental health scales, sleep quality, and psychosocial indicators. Sensor-derived features—such as gait variability, activity regularity, sleep fragmentation, and medication adherence patterns—will be integrated with clinical data to develop supervised machine learning models. Planned approaches include logistic regression, random forests, gradient boosting, and deep learning. Model performance will be evaluated using cross-validation and independent test sets. Primary metrics will include area under the receiver operating characteristic curve, sensitivity, specificity, precision, recall, and *F*_1_-score. Models will be benchmarked against gold-standard clinical diagnoses and validated using temporal subsets of the dataset.

**Results:**

Enrollment for this study started in November 2019 and will continue until March 2030. As of June 2025, we have enrolled 138 participants. Full data analysis has yet to begin.

**Conclusions:**

We aim to develop a reliable and effective sensor system for in-home use that will facilitate the early detection of cognitive and physical decline. In so doing, it will add to our current understanding of digital biomarkers. It is common for older adults to seek clinical intervention only when their cognitive impairment has already reached an advanced stage. The implementation of readily deployable sensor systems within community settings presents us with opportunities for prompt intervention, which holds the potential for delaying or reversing disease progression and allowing for a greater number of functional and meaningful years.

## Introduction

The global population is undergoing significant shifts in its age demographics. By 2050, about 16.3% of the global population will be 65 or older, a sharp increase from 10.0% in 2023 [1]. This shift will pose significant challenges for health care systems due to the economic and medical burdens of providing care for older adults with physical and cognitive limitations. Indeed, frailty and cognitive impairment are two of the most prevalent conditions in the older adult population [2,3].

Frailty, a major syndrome in geriatrics, is characterized by deficits across multiple systems and reduced physiological reserves, leading to increased vulnerability to acute stress and adverse outcomes in older adults. It is associated with a decline in quality of life, heightened disability, mortality, and increased health care use [4]. In high-income countries, the weighted prevalence of frailty was estimated to be 10.7%, whereas the prevalence of prefrailty (PF), a prodromal risk state that can be reversed, was estimated to be 41.6% [5]. Meanwhile, cognitive impairment refers to the deterioration of intellectual abilities such as memory, reasoning, language, and problem-solving. Although it is often observed in older adults, its severity can vary from mild memory lapses to severe dementia. More specifically, mild cognitive impairment (MCI) describes the presence of cognitive decline concomitant with a lack of severity to significantly affect daily functioning [6,7]. MCI affects 6%‐20% of people aged 60‐89 years [8-10], and it is recognized as a prodromal stage of dementia, with a high probability of progression to dementia within 1 to 3 years [11,12].

The combination of early identification and interventions targeting physical, social, and behavioral aspects of MCI and PF can effectively prevent, delay, or even reverse the progression of dementia and frailty, thereby enabling older adults to age well and maintain a good quality of life within their communities [13-24]. Indeed, screening older adults living in the community and in primary care settings more regularly could help in the identification of symptoms of geriatric conditions at an early stage [25-27]. However, the epidemic of dementia in the aging population cannot be tackled using conventional methods, as these either require reliable informant caregivers or sporadic face-to-face screening by primary health care providers [28].

The shortcomings of these conventional procedures have given way to an increasing demand for the use of digital biomarker technologies, some of which can be deployed effectively and continuously in community settings where older adults reside. There has also been a growing emphasis on the ability of these technologies to monitor cognitive function, physical frailty, and mental health [29-32]. A promising approach related to such technologies for monitoring older adults is the use of in-home sensor systems, including passive infrared (PIR) sensors and door contact magnetic sensors. A home-based study investigated the use of PIR motion sensors and acceleration sensors for the continuous monitoring of activities of daily living [33]. This study found that activities of daily living patterns visualized through activity maps could discriminate between older adults with healthy cognition and those with cognitive impairments. Another study demonstrated that a multi-sensor system including PIR motion sensors, magnetic contact sensors, and bed sensors could detect decreased engagement in activities of daily living and increased sleep interruptions in older adults with MCI [26]. Additionally, a study that measured heart rate and gait variability using wearable sensors found that these measures could detect MCI in a cost-effective manner under real-world conditions [34].

Two notable studies conducted by the Collaborative Aging (in Place) Research Using Technology (also known as CART) group used sensors in home-based settings to assess gait speed and variability and identify cognitive decline [35,36]. The first study, which involved 93 participants, revealed that the group with MCI had a coefficient of variation in median walking speed twice that of the healthy group. Over a follow-up period of approximately 2 years, the researchers identified 3 distinct trajectories of mean weekly walking speeds (fast, moderate, and slow) and found that participants with MCI were more likely to be in the slow-speed group. Additionally, the researchers identified 4 distinct trajectories of walking variability, with nonamnestic MCI participants being less likely to be in the stable coefficient of variation group, suggesting that walking speed variability may serve as an early indicator of cognitive decline. In another study conducted over around 2 years, 64 cognitively healthy older adults were observed; those who developed MCI later were more likely to open their pillboxes later in the day and demonstrated more variability in their medication-taking habits than those who remained cognitively stable [37]. Furthermore, a recent meta-analysis revealed that sensors and digital biomarker technologies could detect MCI and frailty with sensitivities of 84% and 82%, respectively [38].

Despite the existing body of research, a systematic review conducted by Piau et al [29] and the work by the Collaborative Aging Research Using Technology group highlighted the scarcity of literature on home-based, real-life evaluations [35,36]. The review also underlined that the samples of extant studies are predominantly homogenous, consisting mainly of Caucasian, urban, educated individuals receptive to technology. In addition, most of the studies used a limited approach (eg, using a single device or technology) and were primarily restricted to participants who lived alone. This proposed study will aim to address these gaps by using various digital biomarker technologies to observe and predict behavioral patterns related to MCI, dementia, PF, and frailty, and differentiate them from those associated with normal aging in older adults living in their homes in Singapore.

This study will focus on using a continuous monitoring sensor system within the homes of older adults (aged 65 years and older) to observe changes in their behavioral patterns over a period of 3 to 6 years. This proposed study extends from a recently completed feasibility study that used a similar home-based sensor setup to monitor behavior patterns in community-dwelling senior citizens in Singapore [26]. The study seeks to distinguish those exhibiting normal aging and those with MCI, early dementia, PF, or frailty and to predict the transition from normal aging to one of these conditions.

To achieve this goal, this study has the following aims:

- To monitor changes in behavior associated with the execution of cognitive-related daily tasks and activities among older adults, and to distinguish between changes attributable to normal aging and those indicative of MCI or early dementia.
- To monitor changes in behavior in performing physical function-related daily tasks and activities among older adults, and to distinguish between changes attributable to normal aging and those indicative of PF or frailty.
- To develop and cross-validate machine learning prediction models derived from clinical data and sensor-based, spatiotemporal, daily activity data that will be used to detect individuals at a high risk of developing MCI, early dementia, PF, or frailty. The performance of these prediction models will also be evaluated against gold standard diagnostic outcomes and cross-validated using subsets of the data.

## Methods

### Ethical Considerations

This study was approved by the SingHealth Centralised Institutional Review Board and registered with the reference number 2019/2026. Written informed consent will be obtained from all participants before enrollment in the study. The study will be conducted in accordance with the Good Clinical Practice guidelines.

### Study Design

This study will use a longitudinal cohort design with 200 Singaporean older adults aged 65 years and older who either reside alone or with others. The follow-up period for the study will span 1 to 6 years, contingent on the continuity of research funding. The daily activities of the participants will be monitored using home-based sensors, in conjunction with annual clinical and cognitive evaluations.

### Sample Size Calculation

As described in the previous sections, an earlier study with 14 participants comparing walking time variability between people with MCI and healthy controls using data obtained continuously from sensors revealed a significant difference between the groups [35]. A subsequent study was conducted by the same research group with 54 and 39 people with healthy cognition and with MCI, respectively, over a mean follow-up time of around 2 years [36]; this subsequent study identified 3 distinct trajectories of mean weekly walking speeds.

Based on these findings on walking speeds and data from the Singapore Longitudinal Aging Study Waves 1 and 2 (unpublished), we conservatively estimate an incidence of MCI or dementia of 30% over a 3-year period. Given the feasibility and budgetary constraints of the study, a sample of 200 participants without dementia (expected 60+ incident cases of MCI or dementia over the study period) will be sufficient to produce a 2-sided 85% CI with a precision of 5%. The sample size calculation is based on CIs for one proportion from a finite population approach via the exact Clopper-Pearson formula and performed using PASS software (version 14; NCSS, LLC) [39].

### Study Eligibility

#### Inclusion Criteria

To be included in the study, participants must (1) be at least 65 years old, (2) have normal cognition or MCI, (3) live independently in the community (ie, no difficulties with any of the instrumental activities of daily living (IADL), see below), and (4) be able to give written informed consent.

#### Exclusion Criteria

Individuals will be excluded from the study if they have a diagnosis of (1) dementia, (2) any other psychiatric disorder, or (3) any condition (eg, frailty, stroke, or Parkinson disease) that results in limitations to perform basic activities of daily living; individuals who are (4) unwilling to adhere to the use of wearables or to have sensors installed in designated areas of their home, or (5) currently participating in a cognitive or motor training intervention trial will be excluded.

### Participant Recruitment and Study Procedure

**Figure 1.**
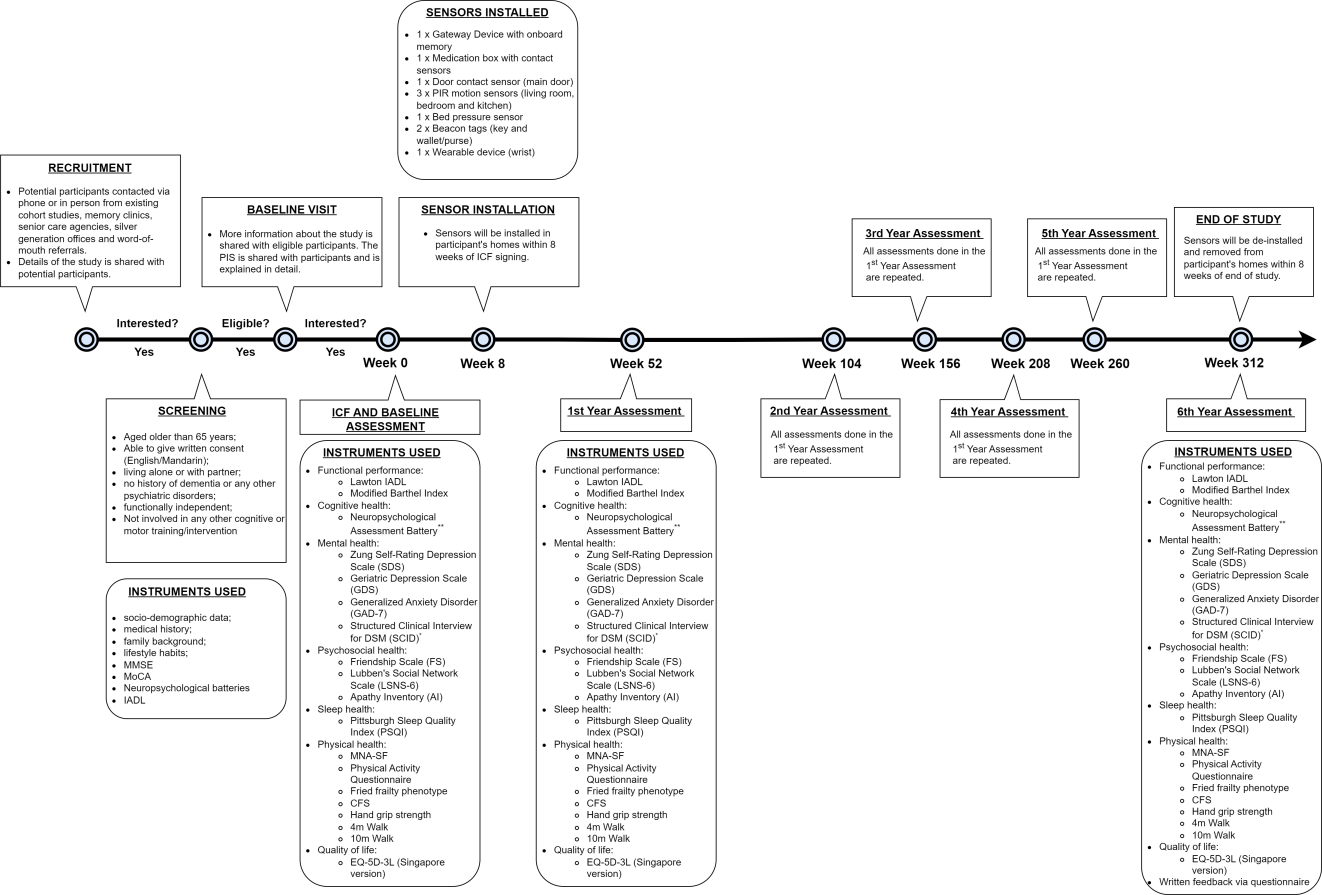
Study design—Timeline and assessments. CFS: Clinical Frailty Scale; IADL: instrumental activities of daily living; MMSE: Mini-Mental State Examination; MNA-SF: Mini Nutritional Assessment Short Form; MoCA: Montreal Cognitive Assessment. *These assessments are only carried out if the participant’s Geriatric Depression Scale score is >5. **The following are included in the neuropsychological assessment battery: (**A**) MoCA, (**B**) MMSE, (**C**) Clinical Dementia Scale (CDR), (**D**) Rey Auditory-Verbal Learning Test (RAVLT), (**E**) Digit Span Test, (**F**) Color Trails Test (CTT), (**G**) Block Design Test, (**H**) Semantic Verbal Fluency (Animals) test, (**I**) Victoria Stroop Test (VST), (**J**) Common Objects Memory Test (COMT).

Participant recruitment will be conducted in various community and hospital settings, including existing cohorts such as the Singapore Longitudinal Aging Study (also known as SLAS), SingHealth Memory clinics, partner senior care agencies, silver generation offices, and word-of-mouth referrals (Figure 1).

Eligible participants who meet the inclusion criteria will be contacted via phone or in person at senior activity centers. Detailed information about the study will be provided to potential participants, allowing them sufficient time to consider their participation and discuss it with their family members. Interested individuals will undergo a baseline visit to provide informed consent and complete the screening procedures. The study will be thoroughly explained to potential participants using a participant information sheet before informed consent is obtained. In cases where a Chinese translation of the participant information sheet is required, a witness will be present to ensure comprehension by the potential participant.

To determine eligibility for participation in the study, trained research personnel will administer various questionnaires and assessments to potential participants. These will include basic demographic and health questionnaires, measures of executive functioning, and measures of cognitive health. These measures will be repeated annually for 3 years.

Upon successful completion of the screening process, eligible participants will be enrolled in the study and undergo further baseline assessments, including assessments of mental health, sleep disturbances, and psychosocial health, which have been associated with cognitive impairment. A brief assessment of medication adherence will also be conducted. All these assessments will be repeated annually. The home-based sensors will be installed after the baseline assessment is completed. Participants may drop out at any point in the study and will be withdrawn if they are diagnosed with dementia.

### Instruments

#### Measure of Functional Performance

The Lawton IADL scale will be used to assess independent functional skills. These skills are considered more complex than basic daily activities and are used to identify current functional levels. The scale comprises 8 domains, each of which ranges in score from 0 (low functioning and dependent) to 4 (high functioning and independent), with the total score ranging from 0 to 23. The scale has been used in older adults as a measure of functioning level and is an accurate tool for screening dementia [40,41]. The convergent and divergent validity of the IADL was tested against known strengths and group differences in Singapore. The IADL as a whole explained 87.6% of the variance. The internal consistency for the physical part of the IADL was 0.91, while 0.78 for the cognitive IADL [41].

The modified Barthel Index (MBI) of Activities of Daily Living will be used to assess the basic activities one needs to be independent at home. It has been widely used in various large-scale studies, including studies conducted in Singapore [42,43]. The scale comprises 10 areas: 6 items on feeding, dressing, bladder, bowel, toilet use, and stair climbing are scored using a 3-point scale (0, 1, or 2 points), 2 items on transferring and mobility on a 4-point scale (0, 1, 2, or 3 points), and the last 2 on bathing and grooming on a 2-point scale. The maximum score is thus 20, and the higher the score, the greater the independence [44]. The MBI has been validated against assessments from occupational therapists for older residents of long-term care facilities. The Kappa coefficient of intra-rater reliability was 0.70‐1.00 for individual tasks; the Kappa coefficients for inter-rater reliability were 0.72‐1.00. Cronbach coefficient alpha of internal consistency was 0.93 [43].

#### Measures of Cognitive Health

The Montreal Cognitive Assessment (MoCA) is a brief screening tool for detecting global cognitive changes in patients with MCI and dementia. The effectiveness of the MoCA in detecting MCI and dementia has been validated in Singapore, and it has also been shown to exhibit diagnostic utility in discriminating between mild and major neurocognitive disorders with an area under the curve (AUC) of 0.77 and 0.99, respectively [45].

The Mini-Mental State Examination (MMSE) is the most common screening tool for the early detection of MCI and dementia worldwide. A localized Singapore version can discriminate between older adults with and without dementia, with a high sensitivity of 97.5% [46]. Normative data have also been determined for Chinese older adults and are widely used in Singapore [47]. With a cut-off point of 26, the MMSE has been shown to have a sensitivity and specificity of 61% and 84%, respectively, and a diagnostic accuracy, based on AUC, of 0.78.

The Clinical Dementia Rating Scale is a semi-structured dementia staging instrument that is completed on a 5-point scale used to characterize 6 domains of cognitive and functional performance. The rating for each domain will be used to calculate a global score between 0 (normal) and 3 (severe dementia) and to determine dementia severity. The scale has been shown to provide reliable and valid assessments for community-dwelling older adults even in the absence of an informant, and there was good agreement between the Clinical Dementia Rating Scale and the clinical assessment status of MCI and dementia (internal consistency =0.82‐0.84; kappa coefficient =0.79) [48].

The Rey Auditory-Verbal Learning Test will be used to evaluate immediate verbal memory, retention of delayed information, learning strategies, proactive and retrospective interference, and delayed recognition. This test has been used to measure immediate and delayed memory performance in older Chinese adults in Singapore [49]. The Rey Auditory-Verbal Learning Test immediate and delayed recall subtests were able to distinguish between converters and nonconverters to dementia with sensitivities of 83% and 85%, respectively, AUCs of 0.75 and 0.76, respectively, and optimal cut-off points of 1 and 2, respectively [50].

The digit span test is part of the Wechsler Adult Intelligence Scale-Fourth Edition and requires participants to read a string of numbers forward (ie, to assess efficiency and capacity for paying attention) and backward (ie, to assess executive function and working memory); its results can show indications of short-term verbal memory. This test has been widely used as a valid tool to discriminate between MCI and dementia among older adults [51], and provided specificities of 99% and 95%, respectively, for MCI and mild dementia, with a cut-off score <4.

The Color Trails Test (CTT) 1 and 2 were designed as culturally appropriate tests in comparison to the Trail Making Test. The CTT 1 requires participants to join 25 numbers in sequence as quickly as possible, whereas CTT 2 requires participants to alternate between colors while joining 25 numbers in sequence. They are used to measure selective attention, processing speed, and cognitive flexibility. Test-retest analysis was performed over a 4-week period to determine the reliability of the CTT, and CTT 1 and CTT 2 showed a Pearson correlation r of 0.90 and 0.91, respectively [52].

The block design test is also part of the Wechsler Adult Intelligence Scale-Fourth Edition, requiring participants to recreate a design shown to them using blocks with different colored sides. The test assesses perceptual reasoning, spatial visualization, and visuo-construction abilities. The block design test has been used for epidemiologic studies among older adults [53], and test performance declines with age across the normative data [54].

The semantic verbal fluency (animals) test will also be administered as a measure of executive function, access to semantic memory, and lexical retrieval. A decline in semantic fluency has been reported as an early indicator of dementia, and it can be used to differentiate the development of dementia from normal age-related cognitive decline [55]. A normative study conducted for the tool showed its reliability, as the number of animals named decreased with an advance in age older than 60 years, and it correlated (*r*=0.52) with orthographic verbal fluency scores [56].

The Victoria Stroop Test will be used to measure response inhibition and cognitive flexibility [57]. This test is divided into 3 conditions and is composed of 24 items. In the first condition, participants are required to name the colors of the dots. In the second condition, they are required to identify the color of the ink each word is printed in (eg, the word Blue is printed in the blue color), while in the last condition, colored words irrelevant to their printed color are presented. The number of errors and the time taken to complete each condition are recorded and scored. The Victoria Stroop Test is reported to be sensitive to both MCI and dementia [58,59].

Age-related memory impairments were assessed using the Common Objects Memory Test (COMT) [60]. The COMT uses pictures of 10 common objects that participants should be familiar with. It involves 3 learning trials with immediate recall and 2 delayed recall trials, one after 5 minutes and another after 30 minutes of distracted activities. The number of objects correctly recalled is scored. The COMT test is not affected by educational levels, language, or ethnicity, and it could be culturally modified to suit the participants. It has been validated in both Caucasian and Asian older adults with dementia [60], and has been included within the Asian Cohort for Alzheimer Disease Data Collection Packet [61].

#### Measures of Mental Health

The Zung Self-Rating Depression Scale and the 15-item Geriatric Depression Scale will be used to determine the level of depressive symptoms among participants. The Zung Self-Rating Depression Scale comprises 20 items that measure depressive symptoms, asking respondents how they felt or behaved in the past week using a 4-point scale ranging from 1 (a little of the time) to 4 (most of the time). A high total score indicates worse depressive symptoms. This scale has been validated among community-dwelling older adults for detecting subsyndromal depression [62]. With cut-off scores of 39 and 40, the scale also showed a sensitivity of 79.2% and an AUC of 0.85 among older adults [63].

The 15-item Geriatric Depression Scale is a shortened form of the original Geriatric Depression Scale and contains 15 questions assessing depressive symptoms over the last 7 days. All 15 items are responded to using a yes-or-no scale, and a score of 5 or above indicates possible depression. This scale showed accuracy in screening for depression among older adults in a meta-analysis, with a pooled sensitivity and specificity of 86% and 76%, respectively, and diagnostic accuracy, based on AUC, of 0.95 [64].

Self-reported anxiety will be captured using the Generalized Anxiety Disorder-7. Participants will respond to 7 questions on how often, over the past 2 weeks, they worried (eg, “Not being able to stop or control worrying,” “Worrying too much about different things”) and experienced somatic tension (eg, “Trouble relaxing”). A 4-point Likert scale response (“0” denoting not at all, while “3” nearly every day) is used. The maximum score is thus 21, while the minimum score is 0, and scores greater than 10 usually suggest a diagnosis of Generalized Anxiety Disorder. This scale has a sensitivity of 89% and specificity of 82% [65]. It has been validated in both cognitively impaired [66] and general populations [67,68], and has been used in Singapore settings [69,70].

#### Measure of Sleep Disturbances

The Pittsburgh Sleep Quality Index is used to measure subjective sleep patterns and quality. It includes 4 open-ended questions and 14 frequency- and semantic-scaled questions that assess sleep quality, latency, duration, disturbances, daytime dysfunction, and sleep efficiency. It can be administered verbally to participants, and a high global score of 5 or more indicates poor sleep. Its suitability for use with older adults in Singapore was demonstrated [71]. It showcased an internal consistency of 0.83 and showed a correlation with greater sleepiness on the Epworth Sleepiness Scale (*r*=0.13) when applied among men [72].

#### Measures of Psychosocial Health

Social vulnerability, such as a lack of social networks, loneliness, and limited social contact, has been shown to predict cognitive decline among older adults [73]. Apathy symptoms are also associated with the conversion from MCI to dementia and incident dementia among community-dwelling older adults [73]. This highlights the importance of considering social networks and apathy when attempting to understand cognitive decline. As such, the 6-item Friendship Scale, Lubben Social Network Scale, and the Apathy Inventory Clinician Version will be used to measure social isolation and apathy.

The 6-item Friendship Scale measures important dimensions contributing to social isolation and connectedness, requiring participants to describe the frequency with which they experience the situations in each question on a 5-point Likert scale ranging from 1 (almost always) to 5 (not at all). The scale has been shown to exhibit concurrent discriminant validity, suggesting its sensitivity to correlates of social isolation among older adults [74]. It has also been validated for use among older adults with a reliability of 0.83 and an excellent internal structure, as the comparative fit index was 0.99 and the root mean square error of approximation was 0.02 [75].

The Lubben Social Network Scale consists of 2 sets of 3 questions aimed at evaluating kinship and nonkinship ties. Total scores range from 0 to 30, with lower scores indicating lower levels of social support. The scale has been validated in past research, showing a Cronbach α of .74 and a convergent validity of −0.3 [76]. It has been used in Singapore to understand social isolation among community-dwelling older adults [77].

The Apathy Inventory clinician version is a scale operationalized into 3 dimensions, namely emotional blunting, lack of initiative, and lack of interest. Items are responded to on a yes-or-no scale to determine the presence or absence of the behavior in the item; then, a 5-point Likert scale ranging from 0 (no problem) to 4 (major problem) is used to determine the score for each dimension. Total scores range from 0 to 12, with scores of 4 and above indicating the presence of apathy. The scale has been validated in older adults and showed a Cronbach α of 0.90 [78]. It has also been applied to determine chronic behavioral changes in Alzheimer disease [79].

#### Measures of Physical Health

Physical frailty will be assessed using the modified Fried frailty phenotype, assessing 5 components: low hand grip strength, slow walking speed, low physical activity, self-reported exhaustion, and low BMI (18.5kg/m^2^) [80]. Those who screen positive for at least 3 of these criteria are considered frail, while those who meet 1‐2 criteria are classified as prefrail. Participants with a Fried score of 0 are deemed to be robust. Exhaustion will be assessed using the Center for Epidemiological Studies-Depression Scale, which asks how often participants felt the statements “everything I did was an effort” and “I could not get going.” An affirmative answer to either or both questions is considered positive for exhaustion. Grip strength will be measured using a hand dynamometer (Jamar Plus+ Digital Hand Dynamometer, USA), with 2 trials for each hand, and the maximum value of 4 trials will be recorded. Gait speed will be assessed using a 10-meter walk at usual pace, with the average of 2 trials recorded. We will adopt the Asian Working Group for Sarcopenia 2019 cut-offs for defining weak hand grip strength and slow gait speed [81]. Physical activity is self-reported using the Physical Activity Vital Sign that quantifies time spent on physical exercise and activities, and values below the lowest quartile from a local community cohort will be deemed physically inactive [82]. The Fried phenotype is the most commonly used of measures for frailty [83]. The frailty phenotype was operationalized using data from the Cardiovascular Health Study, a large-scale study of over 5000 older adults in the United States [80]. It has been used in a number of large-scale longitudinal studies, including one in Singapore, to quantify physical frailty in community-dwelling older adults [84-86].

**Figure 2.**
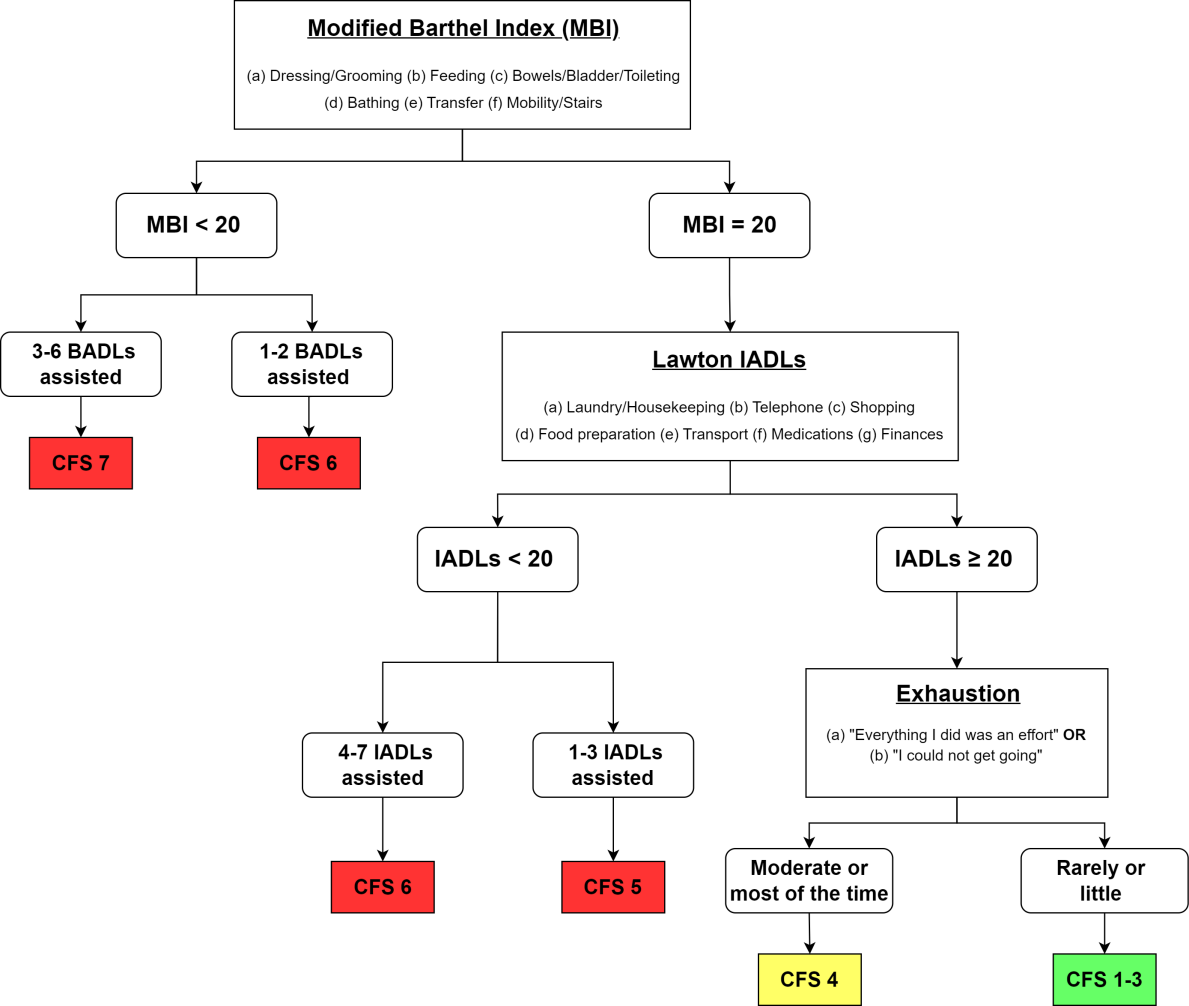
Scoring CFS using the modified classification tree [91,92]. BADL: basic activities of daily living; CFS: Clinical Frailty Scale; IADL: instrumental activities of daily living.

Participants are also scored on the Clinical Frailty Scale (CFS) for a global clinical assessment of frailty status. CFS is a 9-point scale that ranges from 1 (very fit) to 9 (terminally ill) [87,88]. Specifically, CFS has been highly recommended as the instrument of choice for frailty screening in the community and primary care [89]. The CFS has been validated against the Frailty Index in hospitalized older adults in Singapore with an AUC score of 0.91 (95% CI 0.87‐0.95; *P*<.001) [90]. A classification tree had been introduced to facilitate the reliable scoring of CFS [91], and this was modified for our local context (Figure 2) [92]. This method uses information gathered from other validated toolkits—specifically the IADL, MBI, and SF-12 (Exhaustion)—to classify physical frailty. This classification tree approach to CFS will provide us with a more nuanced approach to quantifying frailty in older residents living in the community.

#### Measures of Quality of Life

Health-related quality of life will be captured using the EQ-5D-3L (Singapore version) questionnaire. This questionnaire has 2 parts: the descriptive system and the visual analog scale (VAS). The descriptive system of the EQ-5D-3L has 5 dimensions: mobility, self-care, usual care pain/discomfort, and anxiety/depression. Each dimension has 3 levels of impairment: no problems (level 1), some problems (level 2), and extreme problems (level 3). An EQ-5D summary index (single utility index), which ranges between −1.00 (worst possible health state) and 1.00 (perfect health state), is derived from the 5-digit health state profile using a weighted method. As for the VAS part, it has a visual scale from 0 to 100, with 0 being the “worst health the participant can imagine” while 100 being the “best health the participant can imagine” [93]. The EQ-5D-3L is widely used around the world and has been translated into more than 170 languages. Additionally, it has been validated among patients with chronic noncommunicable diseases [94-96] as well as persons with cognitive impairments. It has been validated against the Health Utilities Index Mark 3 in dementia patients in Singapore [97]. It has been validated against community-dwelling older adults with cognitive impairment [98]. The EQ-5D-3L was validated against corresponding assessment scales: mobility against the Tinetti Index (correlation: 0.371), self-care against the Barthel Index (0.340), usual activities against the Lawton Index (0.628), pain against the VAS pain scale (0.504), and anxiety against GDS (0.703). Reliability (internal consistency) has been reported as 0.69 (Cronbach α).

The Mini Nutritional Assessment Short Form (MNA-SF) is a 6-item questionnaire that has been used to evaluate the nutritional status of older frail adults [99]. It has been used in large cohort studies involving community-dwelling older adults to investigate malnutrition and frailty [100,101]. The MNA-SF comprises 6 questions that cover anthropometric measurements (BMI and unintentional weight loss), diet changes, neuropsychological problems, acute disease, and mobility. Anthropometric measurements are scored between 0 and 3, while all the others range between 0 and 2. The maximum total score for the MNA-SF is thus 14, and a score of less than 7 indicates malnutrition, 8 to 11 indicates a risk of malnutrition, while a score above 12 indicates normal nutritional status [102]. The MNA-SF has been well-validated in a number of large-scale studies involving community-dwelling older adults and has been included in studies that investigated frailty in the Singapore context [99,103-105]. It has been validated against anthropometric measurements and functional status (IADL) in Malaysian older adults—sensitivity: 93.2%, specificity: 79.4% [106].

The 10-meter Walk Test assesses functional mobility and gait speed. Participants will be instructed to walk at their usual (self-selected) walking pace over a distance of 14 meters—2 meters are added at the beginning and at the end of the test to account for the acceleration and deceleration during the test. Participants will walk 3 times—for the first 2, participants will walk without talking, while for the third walk, which is the dual-task gait speed trial, they will be required to count upwards in multiples of 2, starting from the number 16. A handheld stopwatch and a measuring tape will be used to facilitate this test.

### Sensor Setup and Behaviors/Activities Captured

After the baseline assessments are completed, participants will have their homes fitted with a network of sensors (Table 1) for a period of up to 3 to 6 years. Each sensor will regularly monitor the physical environment throughout the day and then wirelessly transmit the collected data to a gateway. The gateway will then send the combined data to the backend server using secure cellular communications for monitoring and processing. A data analytics engine will implement the algorithms for feature extraction of the daily activities of older adults based on the raw sensor data collected by home-based devices. A system monitoring tool will be used to ensure reliable sensor data collection, providing alerts for system downtime. Each data point will only be identifiable through the sensor node identifier, and the mapping between the identifier and location will be securely stored and accessible only to the research project investigators.

The study aims to capture several behaviors of interest using a sensor network. The primary behavior under investigation is forgetfulness, along with in-home activity levels, sleep quality, outings, and physical activity.

**Table 1.** Home-based sensors to be deployed.

Type of sensor	Location of sensor	Data collected	Interpretation
Gateway Device (n=1) (eg, Raspberry Pi 4B + Wi-Fi dongle and Z-Wave stick)	Living room	Consolidation/Storage of all data collected and retransmission to secure	N/A^a^
Passive infrared sensor (n=3) (eg, Aeotec motion sensor 5) for single-occupancy homes only	Living room, bedroom, and kitchen	Presence/absence of activities	Activity levels (in-home/out-of-home/at sensor locations)
BLE^b^ Receivers (n=3) (eg, ESP32) for multi-occupancy homes only	Living room, bedroom, and kitchen	Presence/absence of activities	Activity levels (in-home/out-of-home/at sensor locations)
Door contact sensor (n=1) (eg, Aeotec door sensor 7)	Main door	Number of opening and closing of main house door - per day, per week, and per month	Duration participant spends time at home and out of home
Medication box with contact sensor (n=1) (eg, Aeotec door sensor 7)	Depends on user’s preference	Number of opening and closing of medication box - per day, per week, and per month	Frequency of forgetfulness to take medication
Bed pressure mat + contact sensor (n=1) (eg, Aeotec door sensor 7)	Bedroom	Contact duration	Sleep duration and sleep quality (sleep time, wake time, number of sleep interruptions, and sleep interruption duration)
Beacon tags (n=2) (eg, D15N)	Key and wallet	Locations of key/wallet within the home or out of home	Duration participants spend time at home and out of home. Frequency of forgetfulness to take keys or/and wallet
Wearable sensor (n=1) (eg, Mi Band)	On wrist of participant	Step count and heart rate	Physical activity levels of participant

a Not applicable.

b BLE: bluetooth low energy.

**Figure 3.**
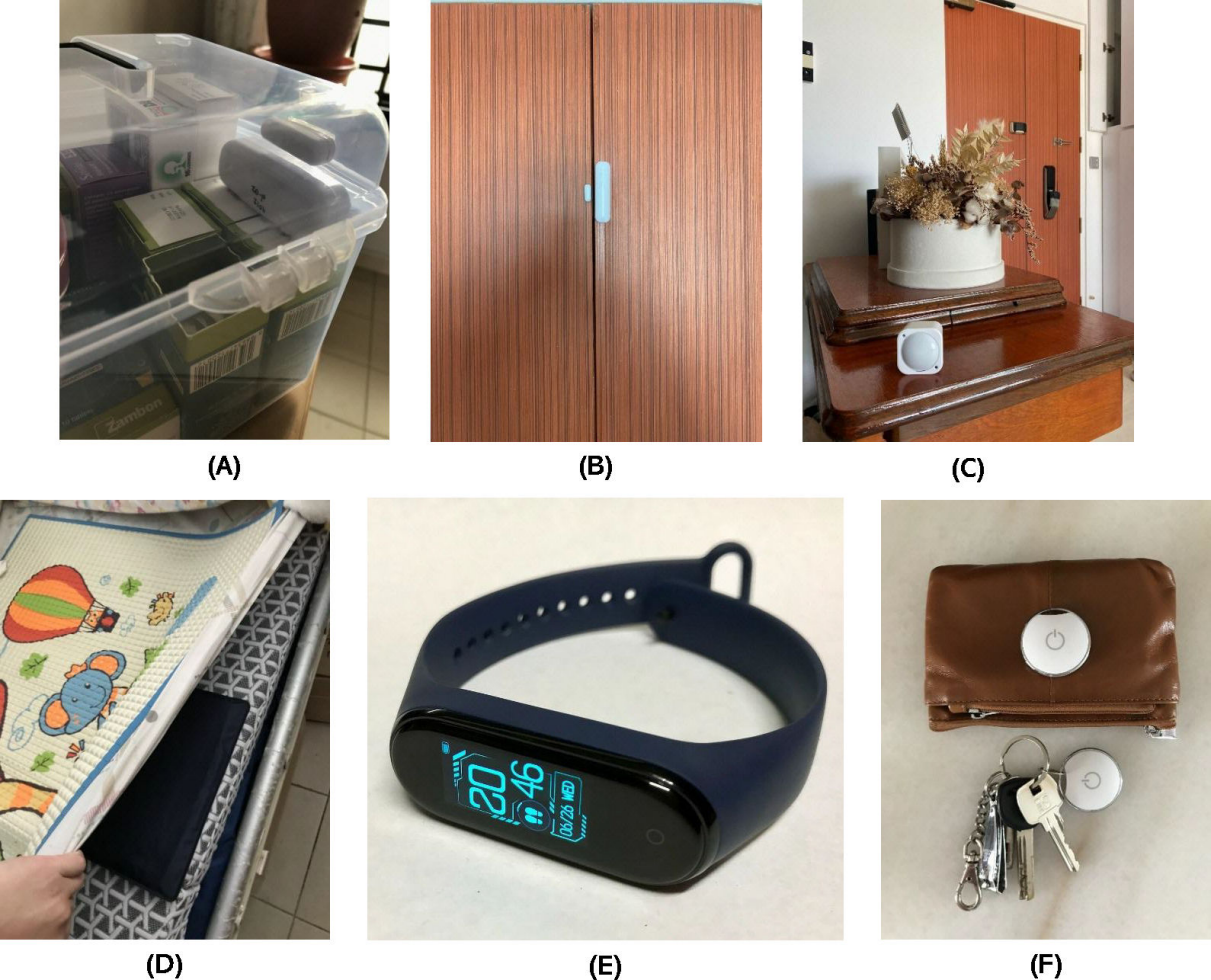
Home-based sensors to be deployed: (A) Contact sensors attached to the medication box. (B) Door contact sensors. (C) Passive infrared sensors. (D) Pressure sensors for beds. (E) Wearable activity band (Xiaomi Mi Band). (F) Proximity sensors on keys and wallets.

To measure forgetfulness, a combination of sensor data will be used. Participants will be provided with a sensor-equipped medication box (Figure 3, top-left) to store their prescription medications. Data will be generated each time the box is opened, and this, in combination with expected medication frequency information obtained at baseline, will enable the determination of the frequency of forgetting to take medication at the prescribed timing. Additionally, medication intake timing variation will be trackable.

In-home activity levels and the frequency of outings for individuals living alone will be inferred using door contact sensors (Figure 3, top-middle) and PIR motion sensors (Figure 3, top-right), which will detect the opening and closing of a participant’s main door. A bed sensor (Figure 3, bottom-left) based on fiber optics technology will be placed under the participant’s mattress to collect data on sleep duration and quality. Participants will be requested to wear a wearable activity band (Figure 3, bottom-middle) to measure heart rate and daily steps. For individuals living with family, the PIR motion sensors will be replaced by 3 signal receivers (such as ESP32) for tracking the location of the individual carrying a wearable or personal beacon. This specific configuration imposes the requirement for additional power points for the 3 USB-powered Bluetooth low energy (BLE) receivers and the participant wearing a smart band or beacon at all times.

Proximity beacons (Figure 3, bottom-right) will be attached to the personal belongings of participants, such as keychains and wallets, allowing for the estimation of the distance between the item and the home gateway. The use of these beacons in conjunction with wearable, PIR motion, and door contact sensors will allow us to determine whether a participant has forgotten to bring these items with them when leaving the home.

All in-home sensors are passive and unobtrusive in nature—they do not require any form of input from the participants. However, future phases of this study could include sensors that might require personalized nudges for the older participants/caregivers. To facilitate this, we will incorporate quick guides and standardize training packages that would be completed by the older participants/caregivers.

To assess frailty, the study will collect data on step count, percentage of sedentary time, percentage of active time, number of outings, movement complexity, and heart rate. Additionally, a new sensor—3DGait (CareCam, Singapore)—will be piloted in this study to capture gait speed during the yearly SPPB assessments. The 3DGait is a Food and Drug Administration–approved innovative software solution that captures human motions using an integrated video acquisition system. It is able to accurately quantify angular, spatial, and temporal parameters related to gait, using a single iPad Pro.

### Privacy and Qualitative Aspects

None of the home-based sensors will have video-recording capabilities. The motion sensors will detect only the presence or absence of motion, and all other captured activities will be inferred through the use of the various sensors mentioned above. Being mindful of the ongoing conversations, which are indeed much needed, in this field of health monitoring and of concerns with intrusiveness, the researchers will elicit feedback from the participants on their reasons for participating in the study, as well as their experiences with remote monitoring systems.

### Outcome Measures and Definitions

The primary outcome measures for this study are the transition of cognitive state (ie, conversion from healthy cognition to MCI or early dementia) and the transition from a robust state to PF or frailty. The secondary outcome measure is global cognitive function deterioration, as indicated by a decline in scores for the MMSE and MoCA, and a composite neuropsychological test battery [107].

MCI and dementia status will be assessed annually through a clinical diagnostic review. An MCI diagnosis will be determined based on the following criteria [108]: (1) self-reported memory and cognitive difficulties or difficulties observed by an informant/clinician; (2) impairment in one or more domains of the MMSE, with a total score ranging from 24 to 27, or a decrease of ≥ 2 points in MMSE scores from baseline; a score 1 to 2 SDs below the age and education-adjusted mean values for at least one neurocognitive domain (ie, attention, memory, executive function, and language or visuospatial abilities), or a decrease of 0.5 SD (vs baseline) during follow-up assessments; (3) a total score of ≥0.5 for the Clinical Dementia Rating Scale; (4) being essentially independent in performing basic activities of daily living; (5) not being diagnosed with dementia.

A dementia diagnosis will require evidence of (1) objective cognitive deficit (ie, a total score for MMSE≤23, or scores for a neurocognitive domain 2 SDs less than the mean values adjusted by age and education); (2) the presence of functional disability (ie, requiring help with at least one basic activity of daily living or a global score for the Clinical Dementia Rating Scale≥1).

Frailty status will be assessed annually using the modified Fried frailty phenotype. Incident PF/frailty will be defined as progression from a state of robustness at baseline to PF/ frailty at follow-up. Progressive frailty will be defined as an incremental Fried score at follow-up relative to baseline.

Additional questionnaires will be performed annually, including the Mini Nutritional Assessment, a Global Physical Activity questionnaire, and a count of the number of near falls/falls over the past year. All assessments, including questionnaires, can be done over separate sessions. Participants have the option to do the assessments at separate sittings if it is tiring for them. However, we will conduct all sessions within a timeframe of 7 days. Additionally, cognitive assessments will be conducted first to avoid physical assessments tiring the participant out.

To mitigate the challenge of high missing and NA rates, daily biomarker values collected from a single participant will be averaged over a weekly or monthly period. The averaging process will produce fewer but higher-quality biomarker records with more reliable values and lower missing/NA rates.

### Data Analysis

#### Sensor Data Processes and Biomarker Extraction

**Figure 4.**
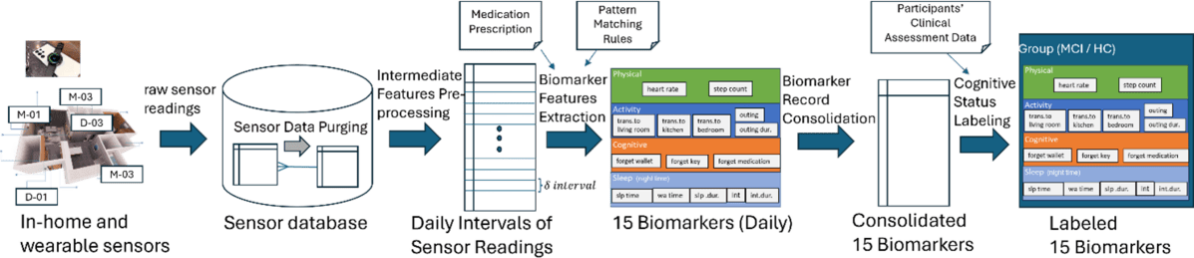
The process of extracting digital biomarkers from the raw sensor data.

Figure 4 shows the process of extracting digital biomarkers from the raw sensor data. The key steps are elaborated below.

- Biomarker feature extraction: Based on the intervals of sensor readings, each daily biomarker feature is extracted following some predefined pattern-matching rules to detect the participant’s state of behavior. For example, to obtain the number of lapses in taking medication (forgetfulness), a rule is used to detect the state that the participant does not take the medication as prescribed based on the absence of readings from the medication box sensor within a range of time intervals in the day.
- Intermediate features preprocessing: Based on the valid sensor data of every participant, each day is divided into intervals of δ minutes (currently, we use δ = 5) in sequential order. The number of sensor readings and their values for every sensor type that occurred within each interval are then recorded.
- Data purging: In this operation, valid raw sensor data are selected based on the periods wherein the in-home sensor gateway system was functional. This step is necessary to filter away unusable sensor data captured when a gateway is down for over a certain period of time.

Daily biomarkers are considered missing when the corresponding behavior states related to the biomarkers cannot be determined. For example, the absence of readings from all the motion sensors during the entire day causes the positional state of the participant to be undetectable, leaving the activity biomarkers of that day missing. Similarly, when the sleeping and awake states cannot be detected because of a prolonged absence of readings from the bed pressure sensor in a day, all sleep biomarkers are considered missing.

On the other hand, biomarker values can be considered as not applicable when the values are not relevant or meaningful under specific circumstances. For example, the sleep time biomarker is considered not applicable when the sleep duration biomarker is 0; forgetting medication is not applicable as well, when the participant does not have any medication prescription.

#### Statistical Analysis

At baseline, descriptive statistics for demographic information and psychometric scores will be analyzed and presented for all participants. Baseline characteristics will be compared between healthy participants and participants with MCI or PF/frailty using 2 independent sample *t*-tests or Wilcoxon rank sum tests (depending on the normality assumption) for continuous variables, and Pearson chi-square tests or Fisher exact tests (where appropriate) for categorical variables.

Group-based trajectory modeling will be used to classify the study population into distinct subgroups based on the patterns of the individual sensor-derived variables and the coefficient of variations recorded over time. The analysis will be conducted via SAS PROC TRAJ (SAS Inc, Cary, NC, USA), which will be used to perform the analyses, a method described by Jones et al [109] in their seminal work. This procedure integrates 2 statistical models estimated simultaneously via maximum likelihood. The first model develops trajectories for the different latent cohorts as a function of time from the baseline. Quadratic or cubic functions of time may be included if they improve model fitness, as determined by the Bayesian information criterion. The second model is a multinomial regression model, which examines the associations of participants’ baseline characteristics with the probability of membership in the homogeneous latent groups. To deal with missing values, complete case analysis is the default method, but SAS PROC TRAJ also allows for the use of multiple imputations to address missingness, where necessary.

#### Artificial Intelligence Predictive Modeling Methods

To detect individuals at high risk of developing MCI/dementia or PF/frailty, machine learning prediction models will be developed and cross-validated according to the extracted clinical data and spatiotemporal daily activity data (from sensors). Model performance (ie, sensitivity, specificity, and AUC) of these prediction models against gold-standard diagnostic outcomes. The models will undergo rigorous cross-validation (CV) to ensure robustness, using subsets of the data to prevent overfitting and ensure generalizability. CV will involve partitioning the data into training and testing sets, enabling the models to be trained on one portion while performance is assessed on another. This approach ensures that the models can reliably predict high-risk individuals across different subsets of the population.

We will apply artificial intelligence (AI) methods, namely spatial-temporal neural network models for predictive modeling, using the sensor-derived data and clinical assessment data to classify older adults’ behavioral change patterns based on the outcomes assessed at the yearly visits across the 3 participant groups (ie, healthy control, MCI/early dementia, and PF/frailty groups). The goal here will be to build predictive models using the longitudinal data regarding specific daily activities (eg, particularly sleep state, heart rate, medication intake, step count, outings, and room transitions) as learning data for the spatiotemporal neural network models, which will then capture the longitudinal dynamics of the participants’ cognitive performance and status.

Two classes of neural network models will be explored and compared, that is, the hierarchical form of self-organizing networks known as fusion adaptive resonance theory (ART) models [110], and deep recurrent neural networks, in particular, long short-term memory [111]. These two neural network models have been shown to provide promising performance in sequence and temporal modeling. For this project, we will formulate the prediction task as a spatial-temporal classification problem, which can typically be addressed via a supervised learning paradigm, wherein sensor-derived data and clinical assessment data collected over a period of time (window) are used as input pattern features, while older adults’ cognitive states (ie, considering the healthy control, MCI, and early dementia groups) are used as the output classes (labels). A key feature of fusion ART models is the ability to perform online one-shot learning, which is advantageous for learning from a small number of training samples. This will be critical in our study because of the technical challenge when using machine learning methods, named as “small data problem,” which is typically faced when working with user data collected from real-world settings. The performance of the fusion ART and long short-term memory–based models will be benchmarked against the commonly used machine learning models and methods, including k-nearest neighbor, support vector machine, decision tree, random forest, and LightGBM.

#### AI Model Training and Validation

The AI-based predictive model will be built using data collected from 200 older adults recruited in this project. Then, considering a period of 3 years and using a sliding prediction window of over 3×365 days, this study can yield up to 1095 daily data points for each participant, together with the cognitive and physical frailty status assessments. In total, we expect to have 2,19,000 daily data points across 200 participants in a 3-year period.

Based on the collected daily data, a CV methodology will be adopted to train and evaluate the performance of the predictive model. For example, using 10-fold CV, 90% of the full dataset will be used to train the model, while the remaining 10% will be used to validate the classification accuracy of the model. The evaluation is repeated 10 times so that all data samples are used for validation once across the 10 experiments. Also, CV evaluations will be further performed in both the cross-sectional (ie, across different participants over the entire time period) and temporal holdout manners (ie, for the entire group of participants over different time periods). Specifically, under cross-sectional CV, data samples from 90% of the participants will be used to train the model, while the data of the remaining 10% participants will be used for validation. This allows us to validate the performance of the trained model on new and unseen participants. For temporal holdout CV, the first 90% data collected over time from all participants are used for training the model, and the last 10% data are used for validation. This enables us to validate the performance of the trained model in predicting the cognitive classes of the existing cohort in the future over time. Standard performance measures for classification will be applied to assess the sensitivity, specificity, and AUC of the predictive models against the gold standard diagnostic outcomes.

CV experiments will be conducted using the data collected in the first 2 to 3 years of the study. Data collected in the subsequent stage of the project can be used as an external validation set for evaluating the predictive models developed.

## Results

Enrollment for this study started in November 2019 and will continue until March 2030. As of June 2025, we have enrolled 138 participants. Full data analysis has yet to begin.

## Discussion

### Principal Findings

We anticipate that continuous in-home monitoring using a multi-sensor system will generate behavioral and functional indicators capable of distinguishing normal aging from early cognitive and physical decline. Based on prior evidence, we expect that sensor-derived features—such as gait variability, activity regularity, sleep patterns, and medication-taking behaviors—will differ meaningfully between individuals who remain cognitively and physically stable and those who progress to MCI, early dementia, PF, or frailty. We further hypothesize that supervised machine learning models integrating these sensor-based features with annual clinical assessments will demonstrate strong predictive performance in identifying individuals at elevated risk of transition. These anticipated findings would support the feasibility of unobtrusive, home-based sensing as a scalable approach for early detection and risk stratification in community-dwelling older adults.

This 6-year longitudinal study will be conducted to monitor changes in behavioral patterns among older adults using a home-based sensor system. Results and learning points from this study will be used to develop a dependable and efficient sensor system for in-home use that can detect early signs of cognitive and physical decline. It is common for older adults in Singapore to seek clinical intervention only when their cognitive impairment has reached an advanced stage. Considering this situation, the implementation of a readily deployable sensor system in community settings presents an opportunity for prompt intervention and holds potential to help delay or even reverse disease progression. The data collected by the sensor system can also be valuably used in supplementing the data from neurocognitive and physical assessments, affording clinicians more reliable information for making a diagnosis. The proposed AI modeling method has several advantages over the methods currently found in the literature. First, the selected behavioral data will be measured using nonintrusive techniques and will be used for building predictive models. While previous studies typically made use of structural neuroimaging or electroencephalogram measurements, our behavioral markers will be collected using simple sensors/wearables and, hence, will be innovative, unobtrusive, and have good potential for upscaling. To our knowledge, this will be a first-of-its-kind study, using longitudinal, sensor-derived behavioral data from people in the community for building predictive models. Second, most prior studies focused on recognizing the cognitive states of participants at a single time point, whereas this study will aim to attempt the more challenging task of predicting the cognitive trajectories of participants over a longer period. If successful, this could contribute to our current understanding of prognostic markers.

This study is planned to evolve into a larger cohort study incorporating serum and neuroimaging biomarkers. We envision that an increasing number of homes will be equipped with clinically studied smart sensors capable of continuously monitoring various health indices related to aging, thereby enabling older adults to enjoy more functional and meaningful lives.

### Anticipated Challenges

#### Privacy

The home-based sensor system that will be used in this study does not have voice or video-recording capabilities. Specifically, PIR sensors can only detect the presence or absence of motion, while the beacon tags lose their connection to the gateway device once they are out of their homes; thus, they do not have tracking capability.

In an earlier feasibility study, there were concerns that the intrusiveness of the continuous monitoring procedures could be an obstacle to recruitment [26]. However, the older adults who lived alone in this cited study expressed enthusiasm to have the system developed to assist them with cognitive monitoring and did not express concerns about intrusiveness, as long as there were no video cameras. Moreover, this study proposal has measures to safeguard data confidentiality. Regardless of these efforts, we will gather qualitative feedback from participants upon study conclusion to better understand the usability and areas for improvement regarding the monitoring system.

#### Maintenance

Since it is anticipated that the sensors need to run smoothly and continuously (24-7) over an extended period of time, we expect that there would be a need to mitigate hardware failures (eg, issues with battery lifespan, faulty hardware), an online dashboard system will be used to monitor the status of all deployed sensors and to promptly alert our data engineers for attention and necessary actions.

Maintenance visits will be scheduled periodically (every 3‐4 months) for battery replacement, when the participant calls to inform about faulty devices, or when the study team determines that the sensors require servicing. Maintenance visits will be arranged as soon as the participant’s schedule permits.

The research team will also provide participants with regular reimbursements to mitigate the electricity costs of the gateway device, which needs to be plugged into an electrical outlet.

#### System Complexity Versus Adherence

Our current sensor configuration offers an elaborate setup, consisting of a home gateway, motion sensors, door contact sensors, medication box, a bed pressure mat, BLE beacons, and wearables. As it may be too complex for low-tech literacy homes to handle and create many failure points over the years, a tiered deployment scheme (such as Base: Gateway+ PIR+door; Plus: Base+ beacons+medbox; and Full: Plus+ bed+wearable) could be considered, whereby the seniors may elect to participate with a preferred level of deployment. Augmented with criteria to upgrade/downgrade per household based on early adherence and living context, this tiered deployment will help to reduce dropouts, wastage in hardware, and effort of deployment/maintenance, while preserving data quality.

#### Wearable Adherence and Contingencies

Long-term wristband use is typically the weakest link of in-home sensing due to various issues such as skin irritation, forgetting, and charging. Guidelines will be provided to the participants regarding predefined minimum wear time KPIs (eg,≥10 h/d on ≥70% of day). We will also provide accessory options, such as spare straps and hypoallergenic bands, and institute regular reminder routines to improve the data quality. Critically, our data processing and AI predictive models will also be designed to tolerate missing wearable-related biomarkers and are still capable of learning a model from readings of other sensors.

#### Medication Sensing Validity

Adherence to consistently taking medications from the designated box is another challenge, as many elders may decant pills into weekly organizers or multiple storage sites. Possible measures to mitigate such risk include briefings to participants and obtaining user acknowledgment during deployment regarding proper use of medication boxes, running calibration against prescriptions, and recording of “not applicable” days operationally.

#### Beacon Practicality and False Positives

The use of key/wallet beacons assumes those items are always attached to the items and carried by the older adults. To mitigate the risk, such as adhesive failures and missing beacons, spare beacons and robust attachments will be provided. In addition, a monthly self-check can be instituted to remind elders of the importance of bringing beacons with them outdoors. To reduce false alarms, the “forgetting” features will be triangulated with the door/PIR patterns and medication timing variability.

#### Multioccupancy Homes and Visitors

Our target expanded cohort will include people both living alone and with others. As the PIR and door sensors are unable to attribute movement to individuals, additional BLE receivers will be installed in place of the PIR sensors to monitor the whereabouts of the specific participants.

#### Data Completeness and Feasibility Endpoints

For measuring data completeness and quality, various operational feasibility outcomes can be tracked and reported quarterly, such as sensor uptime, proportion of analyzable days per participant, mean devices online/day, time-to-alert-triggered maintenance visit, and device-related complaint rates. These metrics will help us assess real-world scalability beyond a research setting.

#### Validation Splits and Label Sparsity

With ≈200 participants but up to 219,000 daily rows over 3 years, cross-validation must avoid leakage. We will replace the generic 10-fold CV with participant-level grouped CV and temporal holdouts (train on early windows, test on later windows). In addition, we will also consider quarterly intermediate labels derived from cognitive trajectories to address annual label sparsity for temporal models.

#### Safety Monitoring and Privacy

To ensure the safety of the older adult participants who live alone, we have incorporated algorithms within our dashboard system to alert the research team if the sensors system does not detect any expected movement (when it was expected, ie, the algorithm “identifies that participant is at home”) for a total of 24 h. Such cases are first checked against the participants’ travel schedule and indoor/outdoor status based on the prior sensor readings. The verified alert cases will be elevated to the research team who will then contact the participant, followed by caregivers/next of kin and community partners to ensure that the participant is well and safe.

#### Data Management

Each participant will be assigned a unique participant identifier number upon successful enrollment. Once assigned, the participant will be followed up anonymously using this unique number. Only the principal investigator and the assigned study team member will have access to the participant data link to the identifiers. All consent forms and hardcopy data collection forms will be stored under lock and key, and in areas of the hospital with restricted access. Data will only be accessed by the principal investigator and the assigned study team member.

All research data will be managed in accordance with Good Clinical Practice standards. Study data will be entered into a secure institutional electronic system that incorporates controlled access and routine backups. Only authorized study personnel will have access according to predefined roles. Administrative files (eg, enrolment logs or scheduling sheets) will be maintained in password-protected spreadsheets stored on an encrypted, access-restricted institutional server. These documents are operational in nature and do not constitute the study’s primary research database.

Three levels of data monitoring will be used to secure data accuracy. At the first level, research assistants will ensure that the data entry and scoring on the hardcopy forms are accurate, and they will also ensure accuracy in the transfer of the hardcopy data to the electronic datasets. At the second level, another research staff member will go through the hardcopy forms and electronic datasets to ensure that the data comply with each other.

#### Protocol Deviations

Serious adverse events and unanticipated problems will be reported within 24 hours to the SingHealth Centralized Institutional Review Board from the day the principal investigator becomes aware of the issue.

#### Dissemination

All findings from this study will be communicated to the scientific community via peer-reviewed publications and conferences. Key findings will be shared with the general public via community workshops and media interviews.

## Supplementary material

10.2196/79490 Checklist 1 STROBE Checklist.

## Abbreviations

AI: artificial intelligence
ART: adaptive resonance theory
AUC: area under the curve
BLE: bluetooth low energy
CFS: Clinical Frailty Scale
COMT: Common Objects Memory Test
CTT: Color Trials Test
CV: cross-validation
IADL: instrumental activities of daily living
MBI: modified Barthel Index
MCI: mild cognitive impairment
MMSE: Mini Mental State Examination
MNA-SF: Mini Nutritional Assessment Short Form
MoCA: Montreal Cognitive Assessment
PF: prefrailty
PIR: passive infrared
VAS: Visual Analogue Scale
