# Supplementary material for: Development and Validation of Machine Learning Models for Predicting Early Cognitive Decline Using Home Sensor–Derived Behavioral Data: Sensors in-Home for Elder Wellbeing (SINEW) Cohort Study
Source: JMIR Res Protoc. 2026 Apr 1;15:e79490. doi: 10.2196/79490 (PMC13043012; doi:10.2196/79490)
Supplement: Checklist 1 [file resprot-v15-e79490-s001.pdf]

STROBE Statement—checklist of items that should be included in reports of observational studies

	Item No	Recommendation
**Title and abstract**	1	(*a*) Indicate the study's design with a commonly used term in the title or the abstract [p.1 (Title), p.1 (Abstract: "longitudinal cohort study")]
		(*b*) Provide in the abstract an informative and balanced summary of what was done and what was found [p.1]
**Introduction**		
Background/rationale	2	Explain the scientific background and rationale for the investigation being reported [pp.1 – 3]
Objectives	3	State specific objectives, including any prespecified hypotheses [p.3 (three aims listed at end of Introduction)]
**Methods**		
Study design	4	Present key elements of study design early in the paper [p.4 ("longitudinal cohort design…1–6 years")]
Setting	5	Describe the setting, locations, and relevant dates, including periods of recruitment, exposure, follow-up, and data collection [pp.4–5 (recruitment sites, Singapore context, dates)]
Participants	6	(*a*) *Cohort study*—Give the eligibility criteria, and the sources and methods of selection of participants. Describe methods of follow-up [pp.4–6]
		(*b*) *Cohort study*—For matched studies, give matching criteria and number of exposed and unexposed [Not applicable]
Variables	7	Clearly define all outcomes, exposures, predictors, potential confounders, and effect modifiers. Give diagnostic criteria, if applicable [pp.14–15 (Outcome Measures), pp.6–13 (clinical instruments), pp.12–13 (sensor-derived behaviors)]
Data sources/ measurement	8*	For each variable of interest, give sources of data and details of methods of assessment (measurement). Describe comparability of assessment methods if there is more than one group [pp.6–13 (all measurement instruments and sensors)]
Bias	9	Describe any efforts to address potential sources of bias [pp.19–21 (anticipated challenges: intrusiveness, adherence, missingness, multi-occupancy, sensor uptime)]
Study size	10	Explain how the study size was arrived at [p.4 (sample size justification using incidence + Clopper–Pearson CI)]
Quantitative variables	11	Explain how quantitative variables were handled in the analyses. If applicable, describe which groupings were chosen and why [pp.14–15 (weekly/monthly averaging), pp.15–17 (biomarker extraction rules)]
Statistical methods	12	(*a*) Describe all statistical methods, including those used to control for confounding [pp.15–17 (trajectory modelling, ML models)]
		(*b*) Describe any methods used to examine subgroups and interactions [p.16 (latent class trajectory groups), p.17 (ML classification groups)]
		(*c*) Explain how missing data were addressed [pp.15–16 (missing biomarker rules; PROC TRAJ imputation)]
		(*d*) *Cohort study*—If applicable, explain how loss to follow-up was addressed [p.5 (withdrawal if dementia diagnosed; annual follow-up)]

		(*e*) Describe any sensitivity analyses [p.17 (temporal holdout CV, cross-sectional CV)]
**Results**		
Participants	13*	(a) Report numbers of individuals at each stage of study—eg numbers potentially eligible, examined for eligibility, confirmed eligible, included in the study, completing follow-up, and analysed [Not applicable – Protocol stage]
		(b) Give reasons for non-participation at each stage [Not applicable – Protocol stage]
		(c) Consider use of a flow diagram [Not applicable – Protocol stage]
Descriptive data	14*	(a) Give characteristics of study participants (eg demographic, clinical, social) and information on exposures and potential confounders [Not applicable – Protocol stage]
		(b) Indicate number of participants with missing data for each variable of interest [Not applicable – Protocol stage]
		(c) *Cohort study*—Summarise follow-up time (e.g., average and total amount) [Not applicable – Protocol stage]
Outcome data	15*	*Cohort study*—Report numbers of outcome events or summary measures over time [Not applicable – Protocol stage]
Main results	16	(*a*) Give unadjusted estimates and, if applicable, confounder-adjusted estimates and their precision (e.g., 95% confidence interval). Make clear which confounders were adjusted for and why they were included [Not applicable – Protocol stage]
		(*b*) Report category boundaries when continuous variables were categorized [Not applicable – Protocol stage]
		(*c*) If relevant, consider translating estimates of relative risk into absolute risk for a meaningful time period [Not applicable – Protocol stage]
Other analyses	17	Report other analyses done—eg analyses of subgroups and interactions, and sensitivity analyses [Not applicable – Protocol stage]
**Discussion**		
Key results	18	Summarise key results with reference to study objectives [pp.18–19 (anticipated findings)]
Limitations	19	Discuss limitations of the study, taking into account sources of potential bias or imprecision. Discuss both direction and magnitude of any potential bias [pp.19–21 (privacy, maintenance, adherence, missing data, multi-occupancy, label sparsity)]
Interpretation	20	Give a cautious overall interpretation of results considering objectives, limitations, multiplicity of analyses, results from similar studies, and other relevant evidence [pp.18–19]
Generalisability	21	Discuss the generalisability (external validity) of the study results [pp.19–20 (scalability, community deployment, future expansion)]
**Other information**		
Funding	22	Give the source of funding and the role of the funders for the present study and, if applicable, for the original study on which the present article is based [p.22–23 (Funding section)]

*Give information separately for cases and controls in case-control studies and, if applicable, for exposed and unexposed groups in cohort and cross-sectional studies.

Note: An Explanation and Elaboration article discusses each checklist item and gives methodological background and published examples of transparent reporting. The STROBE checklist is best used in conjunction with this article (freely available on the Web sites of PLoS Medicine at http://www.plosmedicine.org/, Annals of Internal Medicine at

http://www.annals.org/, and Epidemiology at http://www.epidem.com/). Information on the STROBE Initiative is available at www.strobe-statement.org.
